# OptiSelect and EnShap: Integrating machine learning and game theory for ischemic stroke prediction

**DOI:** 10.1371/journal.pone.0328967

**Published:** 2025-08-13

**Authors:** Pritam Chakraborty, Anjan Bandyopadhyay, Sricheta Parui, Sujata Swain, Partha Sarathy Banerjee, Tapas Si, Hong Qin, Saurav Mallik

**Affiliations:** 1 School of Computer Engineering, Bhubaneswar, Kalinga Institute of Industrial Technology, Bhubaneswar, Odisha, India; 2 Department of Computer Science and Engineering, Jaypee University of Engineering and Technology, Guna, Mohanpur, Madhya Pradesh, India; 3 AI Innovation Lab, Department of Computer Science and Engineering, University of Engineering and Management, Jaipur, Rajasthan, India; 4 School of Data Science, Department of Computer Science, Old Dominion University, Norfolk, Virginia, United States of America; 5 Department of Environmental Health, Harvard T. H. Chan School of Public Health, Boston, Massachusetts, United States of America; 6 Department of Pharmacology and Toxicology, University of Arizona, Tucson, Arizona, United States of America; VIT-AP Campus, INDIA

## Abstract

Stroke analysis using game theory and machine learning techniques. The study investigates the use of the Shapley value in predictive ischemic brain stroke analysis. Initially, preference algorithms identify the most important features in various machine learning models, including logistic regression, K-nearest neighbor, decision tree, support vector machine (linear kernel), support vector machine ( RBF kernel), neural networks, etc. For each sample, the top 3, 4, and 5 features are evaluated and selected to evaluate their performance. The Shapley value method was used to rank the models using their best four features based on their predictive capabilities. As a result, better-performing models were found. Afterward, ensemble machine learning methods were used to find the most accurate predictions using the top 5 models ranked by shapely value. The research demonstrates an impressive accuracy of 92.39%, surpassing other proposed models’ performance. This study highlights the utility of combining game theory and machine learning in Ischemic stroke prediction and the potential of ensemble learning methods to increase predictive accuracy in ischemic stroke analysis.

## Introduction

Stroke is a devastating neurological condition and a leading cause of adult disability, ranking as the second most common cause of mortality worldwide. The majority of strokes are ischemic, caused by blockages in cerebral arteries due to thrombosis or embolism, leading to restricted blood flow and oxygen deprivation in the brain. Without timely intervention, ischemic strokes can result in irreversible neuronal damage, significantly impacting cognitive and motor functions. The urgency of early treatment is well established, as restoring blood flow within the first few hours of stroke onset can dramatically reduce mortality and long-term complications [1]. However, a significant challenge in stroke management is the occurrence of “wake-up strokes," where patients experience a stroke while asleep, delaying intervention and limiting treatment options [2]. This subset of strokes accounts for approximately a quarter of all cases, complicating the ability to administer thrombolytic therapy within the critical therapeutic window. Despite medical advancements, a substantial proportion of stroke survivors continue to face lifelong disabilities, including paralysis, speech impairments, and cognitive decline, necessitating long-term rehabilitation and care [3,4]. Given these challenges, improving early detection and risk stratification methods is paramount to reducing the burden of stroke-related disabilities and fatalities.

Addressing stroke as a major public health concern requires the rapid and accurate differentiation between ischemic stroke (IS) and intracranial hemorrhage (ICH), as the treatment approaches for these conditions differ significantly. Ischemic strokes occur due to obstructed blood flow, whereas ICH results from ruptured blood vessels causing bleeding within the brain [5–8]. Rapid diagnosis is crucial, as treatment strategies such as intravenous thrombolysis or mechanical thrombectomy must be administered within a narrow therapeutic window to maximize recovery potential. Research has shown that timely hospital arrival and intervention within 3 to 5 hours can significantly enhance survival rates and mitigate the extent of brain damage [9–11]. However, achieving rapid diagnosis in real-world settings remains a challenge due to the variability in stroke symptoms and the limitations of traditional risk assessment methods. Existing clinical scoring systems, such as the ${\text{CHA}}_{2}{\text{DS}}_{2}$-VASc score, rely on a fixed set of demographic and clinical parameters, which may not fully capture the complex interactions between risk factors. As a result, there is a growing need for advanced predictive models that can provide more personalized and data-driven risk assessments [12,13].

In recent years, machine learning (ML) and artificial intelligence (AI) have emerged as powerful tools in stroke prediction and diagnosis. Traditional statistical models, though useful, often fail to leverage the full predictive potential of multi-dimensional patient data. To address this limitation, we explore ensemble learning techniques, which combine multiple machine learning classifiers to improve prediction accuracy. In this study, we trained eight machine learning models on a dataset containing key clinical attributes, extracting the most relevant features based on predictive performance. The ranking of classifiers was performed using Shapley values, a game-theoretic approach that quantifies the individual contribution of each feature and model to the overall prediction accuracy. By integrating feature selection and classifier fusion, our approach seeks to optimize ischemic stroke prediction, ensuring that the most informative variables and models are prioritized for final decision-making.

Ensemble learning has gained significant traction in the machine learning and computational intelligence communities due to its ability to reduce overfitting, enhance model robustness, and increase generalizability across different datasets. Initially developed to improve classification accuracy, ensemble methods have evolved to address a variety of real-world challenges, including concept drift learning, error correction, feature selection, incremental learning, and confidence estimation [14,15]. By aggregating predictions from multiple classifiers, ensemble approaches can mitigate the limitations of individual models, leading to a more reliable and stable predictive framework. Recent advancements in fusion methods have further enhanced the efficacy of ensemble learning, with techniques such as stacking, boosting, and bagging proving particularly effective in complex classification problems [16–19]. Given the dynamic and multi-factorial nature of stroke risk assessment, leveraging ensemble-based classification allows for a more nuanced understanding of patient risk profiles, facilitating earlier and more accurate stroke detection.

This paper introduces OptiSelect and EnShap, an innovative framework that integrates game theory principles with machine learning techniques to refine ischemic stroke prediction. The OptiSelect method systematically identifies the most relevant clinical features for stroke risk assessment, ensuring that only the most informative variables are utilized in predictive modeling. Meanwhile, the EnShap classifier employs Shapley values to rank machine learning models based on their contribution to prediction accuracy, thereby optimizing classifier selection. By combining feature selection, model ranking, and ensemble learning, our approach aims to enhance predictive performance, improve interpretability, and support timely intervention in stroke management. This study has the potential to revolutionize stroke risk assessment by bridging the gap between machine learning innovation and clinical applicability, ultimately contributing to improved patient outcomes and more efficient healthcare decision-making.

**Our contributions.**

1. Two novel methods have been created for feature selection and classification for brain stroke detection.
2. Optiselect method has been developed for feature selection.
3. The EnShap classifier was created, where we integrated game theory and machine learning concepts.
4. Discussion of the outcomes using statistical and multi-criteria decision-making techniques.
5. The proposed approaches outperform competitive machine learning models also outperform the other proposed machine learning methods.

The rest of the paper is organized as follows : Section Related work reviews the existing literature. Section Materials and methodology describes the dataset and work flow. Section Results and discussion presents our experimental findings and interpretation. Section Conclusion and future work summarizes the key contributions.

## Related work

Significant approaches have emerged in recent research focused on ischemic stroke prediction: simulated quantum mechanics-based joint learning networks and machine learning models.

Wang *et al*. introduced a simulated quantum mechanics-based joint learning network (SQMLP-net), a groundbreaking method that simultaneously segments stroke lesions and assesses TICI grades. The SQMLP-net achieved remarkable performance metrics with a Dice score of 70.98% and an accuracy of 86.78%. Its innovation lies in its single-input, double-output hybrid network design, effectively integrating segmentation and classification branches. This approach showcased superior outcomes compared to existing methods, demonstrating its potential for accurate stroke lesion segmentation and grading [20].

Another avenue explored in ischemic stroke prediction involves machine learning models, particularly Gradient Boosting Trees (GBT), and their application in assessing stroke risk. Notably, an ensemble model combining GBT and Cox models showcased promising results in identifying individuals at high risk of stroke. This amalgamation displayed the best discrimination and calibration for stroke risk prediction. GBT, in isolation, exhibited high discrimination with AUROC values of 0.833 in men and 0.836 in women for predicting stroke risk over nine years. However, combining GBT and Cox models, the ensemble approach outperformed individual models, boasting higher accuracy, specificity, and positive predictive value. These findings highlight the potential of integrating machine learning, specifically ensemble models, in clinical practice for identifying high-risk individuals susceptible to stroke [21–24].

Moreover, machine learning has been extensively explored for long-term stroke recurrence prediction in ischemic stroke patients. Research studies have utilized various models, including Logistic Regression, Extreme Gradient Boosting, Gradient Boosting Machine, Random Forest, Support Vector Machine, and Decision Tree, employing different sampling strategies. The findings suggest that these machine-learning models exhibit substantial associations with stroke recurrence when integrated with laboratory-based variables. Additionally, these models have showcased stability in stroke recurrence prediction over 1–5 year windows, emphasizing the significance of laboratory-based variables in long-term prognosis. Interpretability and performance were evaluated across six interpretable algorithms, combined with four feature selection strategies, demonstrating the potential of machine learning models in predicting long-term stroke recurrence [25–27].

In the pursuit of improved stroke prediction accuracy, a novel hybrid ML approach has been proposed, specifically tailored for predicting cerebral stroke based on physiological data [28]. This approach successfully reduced the false negative rate, signifying a substantial decrease in misdiagnosis rates related to stroke prediction. Leveraging techniques such as Random Forest regression for imputing missing values and automated hyperparameter optimization based on deep neural networks, this approach showcased considerable promise in enhancing stroke prediction accuracy and reducing the occurrence of false negatives, ultimately contributing to improved patient prognosis [29–32].

A study published in [33] applied three contemporary deep learning approaches to predict 6-month ischemic stroke outcomes using the International Stroke Trial dataset. The deep learning models demonstrated superior performance compared to traditional machine learning methods, underscoring their potential in clinical applications. Research featured in [34] compared the performance of deep learning models to that of neurologists in predicting functional outcomes for large vessel occlusion stroke patients. The study found that while both achieved similar accuracy using clinical data alone, the inclusion of imaging data significantly improved the model’s accuracy to 72%, compared to 64% for neurologists. This suggests that deep learning models, particularly when incorporating imaging data, can enhance predictive accuracy in clinical settings [35].

A study in [36] developed a deep learning-based imaging model combined with clinical variables to predict 90-day stroke outcomes. The model demonstrated reduced subjectivity and user burden, indicating its utility in clinical practice. Research published in the [37] proposed a deep learning network based on Wasserstein Generative Adversarial Networks with Gradient Penalty (WGAN-GP) to generate high-fidelity stroke-positive example data for data augmentation. The results indicated that WGAN-GP effectively extracted key information from stroke samples, enhancing the generalization of the regression network. A predictive analytics approach utilizing machine learning and neural networks has been developed for stroke prediction. This method systematically analyzes various factors in electronic health records to identify the most important predictors of stroke, demonstrating the effectiveness of neural networks in medical data analysis.

Integrating the study of game theory with brain stroke research offers promising avenues for enhancing stroke prediction and treatment strategies. In one instance, while the primary focus of the research was on game-theoretic approaches in machine learning to combat adversarial attacks in cybersecurity applications, an intriguing parallel exists with stroke prediction. Developing robust learning techniques using game theory to counter adversarial attacks aligns with the urgency to anticipate strokes accurately and prevent potential brain damage. Researchers could fortify prediction accuracy by applying similar game-theoretic principles to stroke prediction models, thus preempting strokes and minimizing the neurological impact [38,39].

Moreover, exploring the gamification of machine learning techniques, particularly in object classification scenarios [40], bears relevance to stroke prediction methodologies. Although this paper primarily examines gamification’s role in understanding classifier development, it presents an interesting angle when considering the visualization and interpretation of data related to stroke risk factors. Researchers may unravel critical insights that aid in more accurate predictions and targeted preventive measures by employing gamification principles to analyze and interpret medical data, such as identifying markers for stroke occurrence.

These studies on game theory’s application in machine learning and classifier development offer insightful parallels and methodologies that, when adapted and integrated into stroke prediction research, hold the potential to significantly enhance the accuracy and efficiency of predicting strokes and their subsequent neurological effects.

## Materials and methodology

This section details the dataset used, preprocessing techniques, feature selection methodology, classification approach, and evaluation metrics employed to validate the effectiveness of our proposed framework.

### Dataset

**Fig 1:**
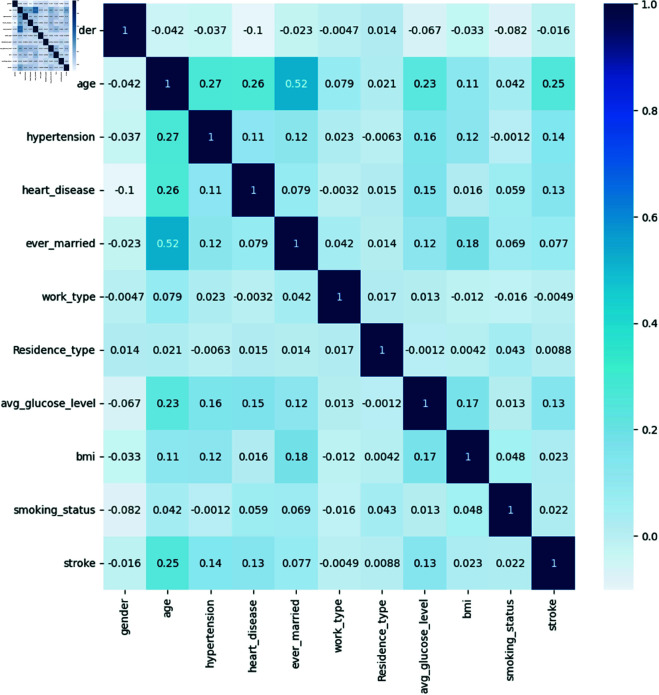
Correlation between features.

This research utilizes the stroke prediction dataset publicly available on Kaggle. The dataset comprises 5110 rows and 12 columns containing specific features relevant to stroke prediction. Table 1 provides a detailed description of each column. Fig 1 shows us the correlation between different dataset features.

**Table 1:** Dataset description.

Feature	Description	Data Type	Non-Null Count
gender	Patient’s gender	object	4981
age	Patient’s age	float64	4981
hypertension	Presence of hypertension (1: yes, 0: no)	int64	4981
heart_disease	History of heart disease (1: yes, 0: no)	int64	4981
ever_married	Marital status (married/not married)	object	4981
work_type	Type of work (Govt_job/Never_worked/Private/Self-employed/children)	object	4981
Residence_type	Residence type (Urban/Rural)	object	4981
avg_glucose_level	Average glucose level	float64	4981
BMI	Body mass index	float64	4981
smoking_status	Smoking status (never smoked/formerly smoked/smokes)	object	4981
stroke	Stroke prediction (1: stroke found, 0: stroke not found)	int64	4981

**Fig 2:**
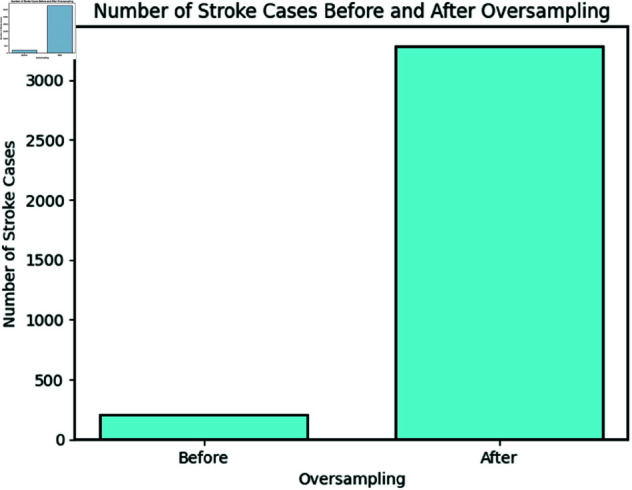
Before and after sampling.

The target variable, ‘stroke‘, is binary, indicating the presence (1) or absence (0) of stroke risk. Analyzing the distribution of this variable reveals a class imbalance, with significantly more instances of non-stroke (0) compared to stroke (1). The dataset undergoes oversampling to address this class imbalance and improve model accuracy. This process increases the number of instances in the minority class (stroke) to match the majority class (no stroke), balancing the data and creating a more representative sample for training and evaluation. The result of oversampling is a dataset with 4861 instances for stroke and no-stroke classes. Fig 2 shows us the difference between the number of stroke classes before and after sampling.

Data imbalance is a common challenge in medical datasets, where the prevalence of certain classes, such as ‘stroke’ cases, is significantly lower than others. This imbalance can lead to biased predictions, with the model favoring the majority class. To address this issue, we employed Synthetic Minority Oversampling Technique (SMOTE), which generates synthetic samples for the minority class by interpolating between existing samples. This approach effectively balances the dataset, resulting in 4861 instances for both the ‘stroke’ and ‘no stroke’ classes.

In addition to SMOTE, we could also explore alternative imbalance mitigation techniques, such as:

- **ADASYN (Adaptive synthetic sampling)**: Unlike SMOTE, ADASYN focuses on generating more samples for minority class examples that are harder to classify, further improving class balance and model performance.
- **Cost-sensitive learning**: Assigning higher misclassification costs to the minority class during model training encourages the model to focus more on correctly classifying underrepresented instances.
- **Ensemble techniques**: Methods such as balanced bagging or boosting create multiple models trained on resampled datasets, ensuring better representation of the minority class across the ensemble.

While the Kaggle dataset provided a valuable resource for developing and validating the proposed framework, it presents certain limitations that warrant consideration. The dataset, being publicly available and preprocessed, may not fully capture the complexities and heterogeneities observed in real-world clinical settings. Key challenges include limited diversity in patient demographics, potential biases in data collection, and the absence of certain clinically relevant features such as longitudinal patient records, genetic markers, or imaging data.

To overcome these limitations, future studies should focus on integrating real-world clinical datasets, which can enhance the robustness and applicability of the proposed framework. These datasets, often collected from hospital networks or health information exchanges, offer several advantages:

**Increased diversity:** Real-world datasets encompass a broader spectrum of patient populations, including diverse ethnicities, age groups, and comorbid conditions, ensuring the model is generalizable across different demographics.

**Rich data sources:** Incorporating clinical datasets that include imaging data, laboratory results, genetic information, and longitudinal records can provide a comprehensive view of patient health, enabling the framework to learn more complex patterns.

**Validation in clinical environments:** Future studies should aim to validate the framework in real-world clinical workflows, assessing its predictive performance, usability, and impact on clinical decision-making in live settings.

**Data privacy and ethics:** Leveraging federated learning approaches or privacy-preserving machine learning techniques can facilitate the use of sensitive clinical data while maintaining patient confidentiality and complying with regulations like HIPAA or GDPR.

#### Geometric mean.

The geometric mean for the ‘STROKE’ class was computed using the formula:

$$\text{Geometric Mean}=\sqrt[{n}]{x_{1}\times x_{2}\times \ldots \times x_{n}}$$ (1)

where: $x_{1},x_{2},\ldots ,x_{n}$ represent the numerical values within the ‘STROKE’ class. *n* denotes the total number of samples in the ‘STROKE’ class.

The resulting geometric mean of approximately 811.96 reveals the average relationship or pattern among the numerical values within the ‘STROKE’ group when multiplied and then taken to the 201st root. This analysis provides insights into the central tendency of the numerical attributes within the ‘STROKE’ category. It offers a comparative perspective on numerical trends between the ‘STROKE’ and ‘NOT STROKE’ classes.

#### Potential biases in the dataset and their impact on real-world deployment.

The dataset used in this study, sourced from Kaggle, may not fully represent the diversity of real-world patient populations. One key limitation is that publicly available datasets often exhibit demographic imbalances, such as an overrepresentation of specific age groups, ethnicities, or geographic regions. This can lead to biased model predictions when applied to broader populations.

For instance, if the dataset primarily includes data from a certain demographic (e.g., middle-aged individuals with access to healthcare), the model may not generalize well to underserved populations, such as elderly patients in rural areas or individuals with limited access to medical services. Additionally, the dataset lacks crucial clinical variables such as genetic markers, lifestyle factors, or socioeconomic status, which could significantly influence stroke risk but are not accounted for in the current model.

These biases could impact real-world deployment by:

- **Reduced Generalizability:** The model may perform well on data similar to the training set but may fail to provide accurate predictions for diverse patient groups.
- **Disparities in Healthcare Outcomes:** If the model systematically underestimates stroke risk in certain populations, it could lead to inadequate preventive measures and increased health disparities.
- **Regulatory and Ethical Concerns:** Bias in AI-driven clinical tools may raise concerns about fairness and equity, potentially affecting acceptance by healthcare professionals and regulatory bodies.

### Proposed OptiSelect feature extraction

Let *X* represent the training dataset with *n* samples and *m* = 10 features:

$$X=[\begin{array}{cccc} x_{1,1} & x_{1,2} & \cdots & x_{1,10}\\ x_{2,1} & x_{2,2} & \cdots & x_{2,10}\\ \vdots & \vdots & \ddots & \vdots \\ x_{n,1} & x_{n,2} & \cdots & x_{n,10} \end{array}]$$ (2)

where *x*_*i*,*j*_ denotes the value of feature *j* in sample *i*.

We train eight models using each dataset feature *X* to predict the target variable.

**Fig 3:**
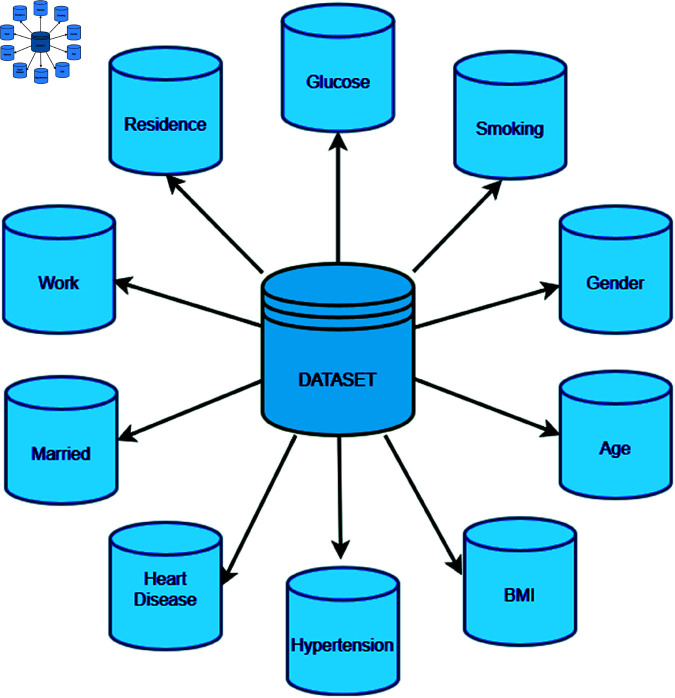
Dataset division.

The feature extraction process has commenced with meticulously segregating each feature alongside the stroke column from the dataset, creating ten distinct datasets, each exclusively containing a singular feature as shown in Fig 3. Following this segmentation, all eight models have been individually trained using the ten datasets, and the achieved accuracies have been systematically recorded, as presented in Table 2.

**Table 2:** Algorithm accuracy comparison for each feature.

Algorithm	Gender	Age	Hypertension	Heart Disease	Married	Work	Residence	Glucose	BMI	Smoking Status
LR	50.6	72.4	51.9	53.2	61.4	57.3	51.7	63.1	56.5	52.9
KNN	89.4	92.1	88.7	88.9	87.9	88.4	88.7	93.6	88.8	88.1
SVM-L	50.6	63.4	52.0	53.2	61.4	56.3	51.8	50.3	49.7	52.8
SVM-R	71.0	73.0	64.4	62.9	69.2	65.3	68.2	58.8	56.8	67.6
NN	72.7	72.4	69.3	66.7	68.7	68.3	67.4	63.0	56.1	70.2
NB	53.0	72.4	54.1	52.3	61.4	56.1	53.9	60.1	60.6	56.5
ADA	60.7	76.4	65.2	66.1	67.5	66.4	66.0	72.6	67.0	64.0
MNB	57.1	61.9	54.1	54.9	61.6	55.6	52.3	59.9	55.2	53.2

After this comprehensive evaluation, all accuracies for each model have been meticulously sorted, and all the accuracies are given in Table 2, leading to a conscientious ranking of features for each model. Specifically, the top 5 features have been identified based on their performance, as depicted in Table 3. A selective approach was then employed to choose the top 3, top 4, and top 5 features for further analysis based on their consistent performance across models.

**Table 3:** Ranking of features for each algorithm.

Algo	Gender	Age	Hypertension	Heart Disease	Married	Work	Residence	Glucose	BMI	Smoking Status
LR	10	1	8	6	3	4	9	2	5	7
KNN	3	2	6	4	9	7	6	1	5	8
SVM-L	8	1	6	4	2	3	7	9	10	5
SVM-R	2	1	7	8	3	6	4	9	10	5
NN	2	1	4	7	5	6	7	9	10	3
NB	9	1	7	10	2	6	8	4	3	5
ADA	10	1	8	6	3	5	7	2	4	9
MNB	4	1	8	7	2	5	10	3	6	9

#### Top 3 features.

**Fig 4:**
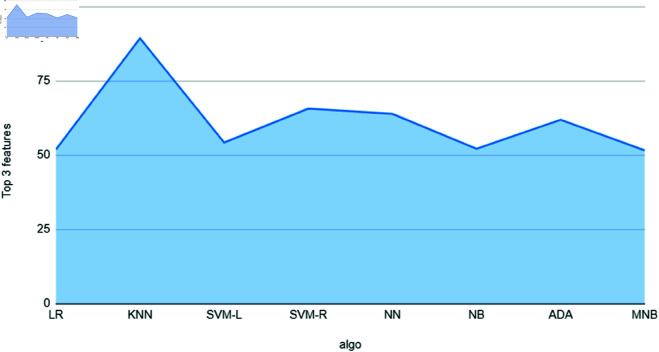
Accuracy of all models with top 3 features.

In this section, we focused on discerning the top three features associated with different machine learning models; for instance, KNN exhibited substantial reliance on heart disease, glucose, and age as pivotal features, while LR emphasized Age, Glucose, and Marital Status (Married) as influential factors. SVM-L and SVM-R highlighted features like heart disease, marital status, work-related factors, age, gender, and glucose levels, indicating their impact on model accuracies. Additionally, NB identified age, marital status, and glucose as primary determinants. NN showed importance in gender, age, and hypertension, ADA in age, marital status, and glucose, and MNB in gender, age, and marital status. Accompanying this feature identification, the algorithm accuracies have been determined which is also shown in Fig 4, portraying LR with an accuracy of 51.896%, KNN with 89.381%, SVM-L with 54.22%, SVM-R with 65.665%, NN with 63.885%, NB with 52.142%, ADA with 61.885%, and MNB with 51.589%. These accuracy assessments denote the individual performance levels achieved by the respective algorithms.

#### Top 4 features.

**Fig 5:**
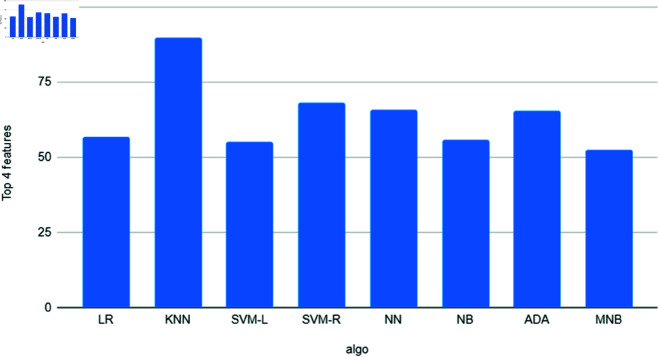
Accuracy of all models with top 4 features.

We have used the top four features of various machine learning models here. Each model was again trained with these four attributes, attaining elevated accuracies across all models examined, as shown in Fig 5. K-Nearest Neighbors (KNN) presented notable features including ‘heart disease’, ‘glucose’, ‘age’, and ‘gender’ and attained an accuracy of 89.823%. Logistic Regression (LR) achieved an accuracy of 56.89%, which includes ‘Age’, ‘Glucose’, ‘Married’, and ‘Work’ as influential attributes. The Support Vector Machine with Linear Kernel (SVM-L) obtained 55.057% highlighted ‘heart disease’, ‘married’, ‘work’, and ‘age’ as crucial factors. Meanwhile, the Support Vector Machine with Radial Kernel (SVM-R) has trained with ‘age’, ‘gender’, ‘married’, and ‘residence’ and got 67.889% accuracy. Naive Bayes (NB) underscored ‘age’, ‘married’, ‘glucose’, and ‘BMI’. For Neural Networks (NN), ‘gender’, ‘age’, ‘hypertension’, and ‘smoking’ emerged as influential factors. Additionally, ADA Boost identified ‘age’, ‘married’, ‘glucose’, and ‘BMI’. In contrast, Multinomial Naive Bayes (MNB) pinpointed ‘gender’, ‘age’, ‘married’, and ‘glucose’ as pivotal attributes, and NN displayed 65.55%, NB yielded 55.689%, ADA recorded 65.297%, and MNB reached 52.339% of accuracy. We can see that the accuracy of all the models with four features outperformed all models with only three features.

#### Top 5 features.

**Fig 6:**
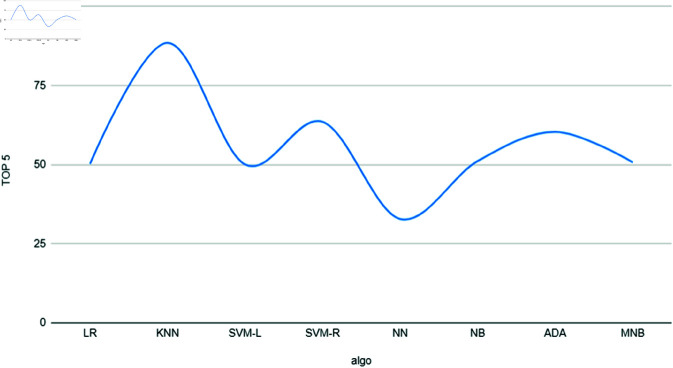
Accuracy of all models with top 5 features.

Several salient features of the machine learning models under investigation were revealed: Support Vector Machine with Linear Kernel (SVM-L) emphasized ‘heart disease’, ‘married’, ‘work’, ‘age’, and ‘smoking status’; K-Nearest Neighbors (KNN) displayed ‘heart disease’, ‘glucose’, ‘age’, ‘gender’, and ‘BMI’; Logistic Regression (LR) revealed ‘Age’, ‘Glucose’, ‘Married’, ‘Work’, and ‘BMI’; Support Vector Machine with Radial Kernel (SVM-R) identified ‘age’, ‘gender’, ‘married’, ‘residence’, and ‘smoking status’; Naive Bayes (NB) highlighted ‘age’, ‘married’, ‘glucose’, ‘BMI’, and ‘smoking status’; Neural Networks (NN) depicted ‘gender’, ‘age’, ‘hypertension’, ‘smoking’, and ‘married’; ADA Boost identified ‘age’, ‘married’, ‘glucose’, ‘BMI’, and ‘work’; and Multinomial Naive Bayes (MNB) disclosed ‘gender’, ‘age’, ‘married’, ‘glucose’, and ‘work’. As a result, the accuracy for LR was 50.253%, KNN was 88.496%, SVM-L was 50.063%, SVM-R was 63.527%, NN was 32.842%, NB was 51.152%, and ADA was 60.396% and for MNB it was 50.725% given in the chart shown in Fig 6.

**Fig 7:**
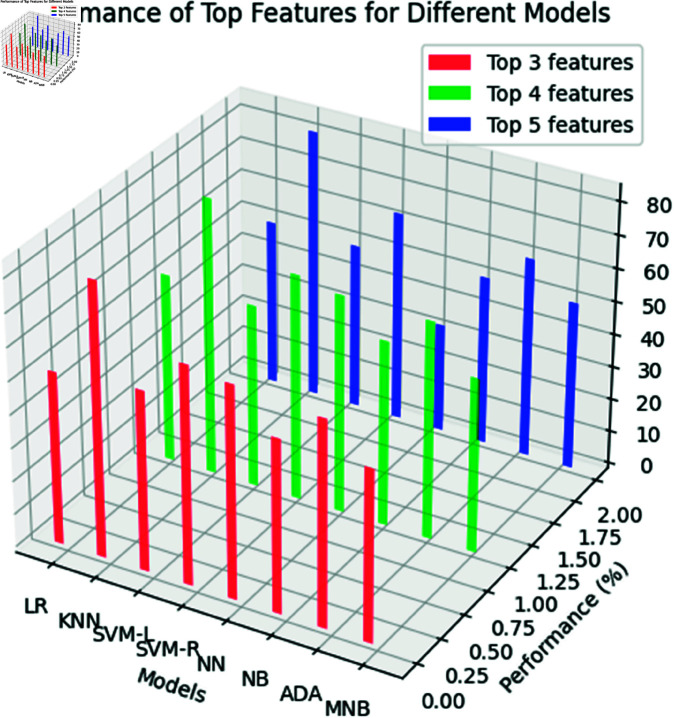
Accuracy of models with top features.

**Fig 8:**
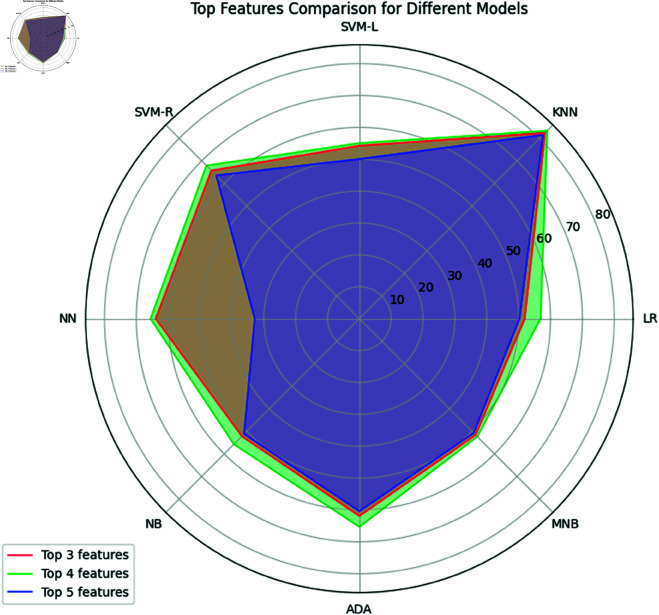
Accuracy of models with different number of features.

**Fig 9:**
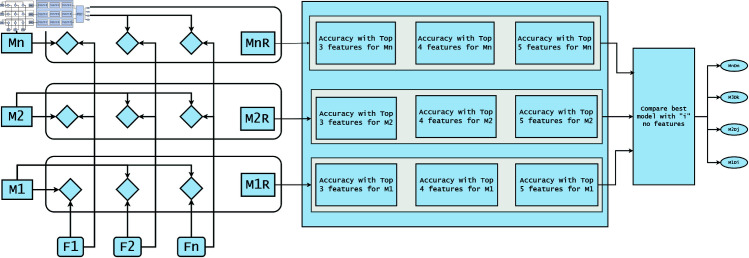
OptiSelect feature extraction.

The accuracies of all models with different features have been compared in Fig 7. It is very clear from the result that all the models show great accuracy when trained with their top 4 features, but when we introduce one more feature, i.e., the top 5 features, we see that all models’ accuracy has been reduced. We have also seen that when we remove one feature from the top 4 features and give the models the top 3 features, they also get less accuracy than accuracies with the top 4 features. All the results are shown in Table 4 along with a graphical representation of accuracy in Fig 8. The OptiSelect feature extraction method diagram is given in Fig 9. We have described our proposed feature selection method, i.e., OptiSelect in Algorithm 1.

**Table 4:** Algorithm comparison - top performances.

Algorithm	3 features (%)	4 features (%)	5 features (%)
LR	51.896	**56.89**	50.253
KNN	82.381	**83.396**	81.496
SVM-L	54.226	**55.057**	50.063
SVM-R	65.665	**67.889**	63.527
NN	63.885	**65.55**	32.842
NB	52.142	**55.689**	51.152
ADA	61.885	**65.297**	60.396
MNB	51.589	**52.339**	50.725

### Proposed EnShap classification

In this paper, for classification, we have used the concept of Shapley value from game theory, which helped us pick the suitable classifier for further processing. Additionally, we have used the concept of Ensemble learning from the field of machine learning, so in our proposed classification, we have integrated the two great fields of science, i.e., Machine learning and Game theory.

**Algorithm 1. Proposed OptiSelect feature selection algorithm.**

1: **Input:** Dataset with features **X** and stroke column **y**

2: **Output:** Selected top features ${𝐗}_{\text{selected}}$ for further analysis

3: **procedure** Feature_Extraction

4:   **for** each feature *F*_*i*_ in **X do**

5:    **for** each subset *S*_*j*_ of data **do**

6:     ${𝐗}_{\text{subset}}\leftarrow$ CreateSubset$𝐗,F_{i}$

7:    **end for**

8:   **end for**

9: **end procedure**

10: **procedure** Model_Training

11:   **for** each dataset ${𝐗}_{\text{subset}}$ **do**

12:    **for** each model *M*_*k*_ **do**

13:     $M_{k}\leftarrow$ TrainModel${𝐗}_{\text{subset}}$

14:     Record accuracy $A_{k,\text{subset}}$ achieved by *M*_*k*_

15:    **end for**

16:   **end for**

17: **end procedure**

18: **procedure** Accuracy_Sorting

19:   Sort accuracies $A_{k,\text{subset}}$ obtained for each model *M*_*k*_ across datasets

20:   Create a table displaying sorted accuracies for each model

21: **end procedure**

22: **procedure** Feature_Ranking

23:   **for** each model *M*_*k*_ **do**

24:    Identify top-performing features based on accuracies

25:    Determine ${𝐗}_{\text{top\_5}}$ - top 5 features for *M*_*k*_

26:   **end for**

27: **end procedure**

28: **procedure** Top_Feature_Selection

29:   Select ${𝐗}_{\text{top\_3}}$, ${𝐗}_{\text{top\_4}}$, and ${𝐗}_{\text{top\_5}}$ based on consistent performance across models

30: **end procedure**

31: **procedure** Retraining_With_Selected_Features

32:   **for** each selected feature set ${𝐗}_{\text{selected}}$

33:    RetrainModels${𝐗}_{\text{selected}}$

34:    Record performance metrics achieved by each model with selected features

35:   **end for**

36: **end procedure**

37: **function** Create_Subset$𝐗,F_{i}$

38:   Segregate data in **X** based on feature *F*_*i*_ into ten distinct datasets

39:   **return** ${𝐗}_{\text{subset}}$

40: **end function**

41: **function** Train_Model${𝐗}_{\text{subset}}$

42:   Train a model using dataset ${𝐗}_{\text{subset}}$

43:   **return** Trained model

44: **end function**

45: **procedure** Retrain_Models${𝐗}_{\text{selected}}$

46:   Retrain models using selected top features ${𝐗}_{\text{selected}}$

47: **end procedure**

48: Call procedures: FeatureExtraction, ModelTraining, AccuracySorting, FeatureRanking, TopFeatureSelection, RetrainingWithSelectedFeatures

#### Shapley.

The Shapley value, a vital concept in cooperative game theory, enables equitable allocation of the combined payoff generated by a coalition of players; Shapley values were named in honor of Lloyd Shapley, who introduced the concept in 1951. In our paper for ranking classifiers, the Shapley value serves as a tool to assess the significance of individual classifiers within an ensemble. For a coalition game defined by a characteristic function v, the Shapley value assigned to the player i is calculated using the formula:

$$\phi_{i}(v)=\sum_{S\subseteq N⧵\{i\}}\frac{|S|!(|N|-|S|-1)!}{|N|!}\left(v(S\cup \{i\})-v(S)\right)$$ (3)

Breaking down the elements within this equation pertinent to classifier ranking:

- $\phi_{i}(v)$ represents the Shapley value attributed to classifier *i*, derived from the characteristic function *v*.
- *N* signifies the complete set of classifiers involved.
- *S* denotes a coalition of classifiers excluding classifier *i*.
- v(S∪{i}) denotes the performance or contribution of the coalition including classifier *i*.
- *v*(*S*) indicates the performance or contribution of the coalition excluding classifier *i*.
- |S| signifies the number of classifiers within coalition *S*.
- |N| represents the total count of classifiers in the ensemble.

Imagine a group of friends playing a team-based game where each player contributes differently to the team’s success. Some players are highly skilled and have a big impact, while others contribute less but still play a role. The question is: how do we fairly distribute the team’s total winnings among the players based on their contributions?

The Shapley value answers this by considering all possible ways players could have joined the team and measuring how much each individual improved the team’s performance in every possible scenario. It averages these contributions to assign a fair value to each player.

In our case, we treat machine learning classifiers as “players" in an ensemble and evaluate how much each classifier improves prediction accuracy when added to different combinations of models. This helps us rank and select the most valuable classifiers for better stroke prediction. Similarly, we use the Shapley value to rank input features based on their importance in making predictions.

By using this approach, our model ensures that the most influential features and classifiers receive appropriate weight, leading to improved accuracy and interpretability.

Utilizing the Shapley value facilitates the assessment of the average marginal contribution of each classifier across all potential coalitions they participate in, considering different arrival sequences. This assessment enables a fair evaluation of the classifiers’ contributions to the ensemble’s overall performance, allowing us to rank them accordingly. The algorithm for Shapley value calculation for classifiers is mentioned in Algorithm 2. The Shapley values based on accuracies for the chosen classifiers are as follows: **KNN** has a Shapley value of **0.1307**, **LR** has a Shapley value of **0.1235**, **SVM-L** has a Shapley value of **0.1231**, **SVM-R** has a Shapley value of **0.1259**, **NB** has a Shapley value of **0.1233**, **ADA** has a Shapley value of **0.1254**, **MNB** has a Shapley value of **0.1225**, and **NN** has a Shapley value of **0.1254**.

**Algorithm 2. Shapley value calculation for classifiers.**

**Input:** List of classifiers, List of accuracies for each combination of classifiers

**Output:** Shapley values for each classifier

1: **procedure**Calculate_Shapley_ValuesClassifiers, Accuracies

2:   N← Number of classifiers

3:   Initialize Shapley values for each classifier: SV←[0,0,…,0]

4:   **for** i←1 **to** *N* **do**

5:    **for** S **in** all subsets of Classifiers excluding i **do**

6:     m←|S|

7:     contribution←Accuracies[S∪{i}]−Accuracies[S]

8:     $SV[i]\leftarrow SV[i]+\frac{contribution}{m\times (N-m+1)}$

9:    **end for**

10:   **end for**

11:   Normalize Shapley values: $SV\leftarrow SV\times \frac{1}{N}$

12:   **return** Shapley values *SV*

13: **End procedure**

**Given:** List of classifiers *Classifiers*, Accuracies for each combination of classifiers

**Call:** Calculate_Shapley_Values(Classifiers, Accuracies)

#### Ensemble.

This section employs the ensemble classification methodology to optimize model selection based on the Shapley values derived from normalized accuracies. The top five classifiers, determined by their respective Shapley values, are utilized for ensemble modeling. The classifiers selected include K-Nearest Neighbors (KNN), Logistic Regression (LR), Support Vector Machine with Linear Kernel (SVM-L), Support Vector Machine with Radial Kernel (SVM-R), Naive Bayes (NB), AdaBoost (ADA), Multinomial Naive Bayes (MNB), and Neural Network (NN).

Moreover, to enhance the performance of these classifiers, OptiSelect feature selection techniques have been employed to identify the most influential features for each classifier. The four most discriminative features for each of the top five classifiers, obtained through OptiSelect feature selection, are outlined in Table 5.

**Table 5:** Selected models and their Highest preferred features.

Algorithms	Feature 1	Feature 2	Feature 3	Feature 4
KNN	Age	Glucose	Heart disease	Gender
LR	Age	Glucose	Married	Work
SVM-L	Heart disease	Married	Work	Age
SVM-R	Age	Gender	Married	Residence
NB	Age	Married	Glucose	Bmi

The selection of these specific features for each classifier is based on their significance in contributing to the overall predictive performance, as identified through rigorous feature selection techniques.

#### Enshap model.

The Enshap Classifier algorithm begins by processing a dataset 𝒟 containing features **X** and corresponding labels **y**. This algorithm comprises multiple key steps to create an ensemble model ℰ that leverages the strengths of diverse models and important features.

Initially, the algorithm undergoes a Feature Selection and Model Training phase. For each of the eight diverse models (*M*_*i*_) within ℳ, it identifies the four most relevant features ${𝐅}_{i}$ by employing specific feature selection techniques tailored to each model’s characteristics. Subsequently, these selected features are used to train the respective models (*M*_*i*_).

**Algorithm 3. Proposed Enshap classifier algorithm.**

1: **Input:** Dataset 𝒟 with features **X** and labels **y**

2: **Output:** Ensemble model ℰ using top 5 models and their 4 most important features

3: **procedure** Feature_Selection_And_Model_Training

4:   **for** each model *M*_*i*_ in ℳ, where ℳ represents 8 diverse models **do**

5:    ${𝐅}_{i}\leftarrow$ Select_Features **X**, **y**, *M*_*i*_

6:    $M_{i}\leftarrow$ Train_Model${𝐅}_{i}$

7:    Evaluate model accuracy *A*_*i*_ on the test set

8:   **end for**

9: **end procedure**

10: **procedure** Shapley_Value_Calculation

11:   **for** each combination *C* of models **do**

12:    Acc(C)← CombineModels*C*, **X**, **y**

13:   **end for**

14:   **for** each model *M*_*i*_ **do**

15:    Calculate Shapley value *S*_*i*_ based on Acc(C) considering all *C* containing *M*_*i*_

16:   **end for**

17: **end procedure**

18: **procedure** Model_Ranking

19:   Rank models based on their Shapley values *S*_*i*_

20:   Select top 5 models $M_{\text{top\_5}}$ with highest *S*_*i*_

21: **end procedure**

22: **procedure** Ensemble_Model_Creation

23:   **for** each selected model *M*_*j*_ in $M_{\text{top\_5}}$ **do**

24:    ${𝐅}_{\text{imp}}\leftarrow$ Retrieve_Important_Features*M*_*j*_

25:   **end for**

26:   Construct ensemble model ℰ using top 5 models $M_{\text{top\_5}}$ and their 4 most important features ${𝐅}_{\text{imp}}$ for prediction

27: **end procedure**

28: **function** Select_Features **X**, **y**, *M*_*i*_

29:   Identify 4 optimal features using the feature selection method based on model *M*_*i*_

30:   **return** Selected 4 features ${𝐅}_{i}$

31: **end function**

32: **function** Train_Model${𝐅}_{i}$

33:   Train a model using selected features ${𝐅}_{i}$

34:   **return** Trained model *M*_*i*_

35: **end function**

36: **function** Combine_Models*C*, **X**, **y**

37:   Combine models in set C and compute combined accuracies Acc(C)

38:   **return** Acc(C)

39: **end function**

40: **function** Retrieve_Important_Features*M*_*j*_

41:   Extract 4 most influential features for model *M*_*j*_

42:   **return** ${𝐅}_{\text{imp}}$

43: **end function**

44: Call procedures: FeatureSelectionAndModelTraining, ShapleyValueCalculation, ModelRanking, EnsembleModelCreation

Following this, the algorithm proceeds with Shapley Value Calculation, where it evaluates the impact of each model on the ensemble’s accuracy. The Shapley value *S*_*i*_ of a model *M*_*i*_ is computed by assessing the change in accuracy when *M*_*i*_ is added to various combinations of models, considering all possible combinations.

After computing Shapley values, the models are ranked based on their respective values. The top 5 models with the highest Shapley values are selected, forming the set $M_{\text{top\_5}}$. These top models are considered for Ensemble Model Creation. For each model *M*_*j*_ in $M_{\text{top\_5}}$, the algorithm retrieves the 4 most important features ${𝐅}_{\text{imp}}$ associated with that model.

Finally, the ensemble model ℰ is formed by combining predictions from the top 5 models ($M_{\text{top\_5}}$) using their respective important features. The ensemble model ℰ aggregates the predictions by employing a weighted average based on the selected models and their significant features.

**Fig 10:**
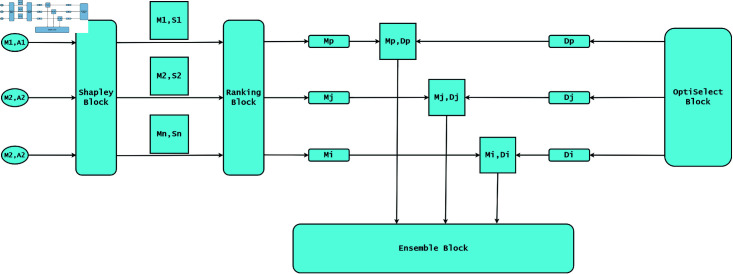
Proposed EnShap model.

**Fig 11:**
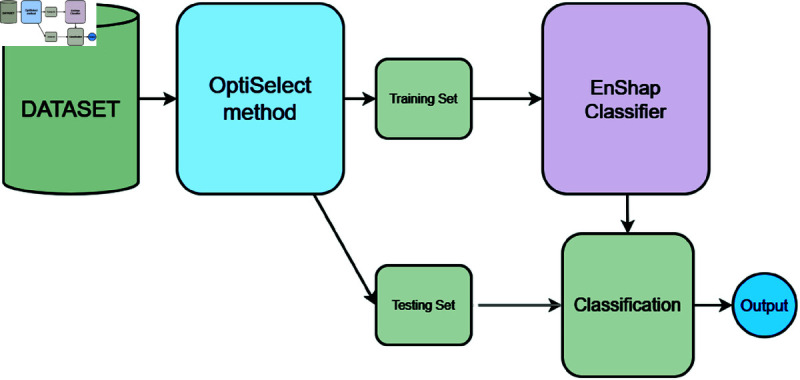
Flow chart of proposed method.

The Enshap Classifier algorithm employs a systematic approach that integrates OptiSelect feature selection, model training, Shapley value computation, model ranking, and ensemble model creation. This method aims to construct an ensemble model ℰ capable of achieving improved predictive accuracy by harnessing the expertise of diverse models and their key features in the classification process. We have described our proposed classification method, i.e., EnShap classifier in Algorithm 3, and a detailed diagram of our proposed classifier is given in Fig 10. the end to flow diagram of our method is shown in Fig 11.

### Hardware used

The implementation of the proposed framework was carried out on Google Colaboratory, utilizing its cloud-based computational environment. The system was equipped with 12.7 GB of RAM, 15.0 GB of GPU memory, and 78.2 GB of available disk space, providing sufficient resources for training and evaluation.

## Results and discussion

In our study on integrating game theory and machine learning for ischemic stroke prediction, we conducted evaluations comparing predictions with ground truth data using specific metrics. The dataset consists of patient-related features pertinent to stroke prediction, including gender, age, hypertension, heart disease history, marital status, work type, residence type, average glucose level, BMI, smoking status, and stroke prediction.

To assess the predictive model’s performance, we employed multi-class confusion matrices to analyze the predictive accuracy for three distinct classes: ‘core,’ ‘penumbra,’ and ‘healthy brain.’ Here, we define:

- **TPc (True Positive)**: Represents accurate predictions of a particular class (e.g., ‘core’).
- **FPc (False Positive)**: Denotes instances where the model falsely predicts a class (‘core’) not present in the ground truth.
- **FNc (False Negative)**: Indicates the model’s failure to predict a class (‘core’) that exists in the ground truth.
- **TNc (True Negative)**: Refers to accurate rejections of a class (‘core’) that does not exist in the ground truth.

Each entry in the multi-class confusion matrix reflects the count of instances based on the defined classes and their predictions from the model.

From these confusion matrices, we derive the following metrics for each class c∈C:

- **Recall (recc)**:
  $$\text{recc}=\frac{TPc}{TPc+FNc}$$ (4)
- **Precision (precc)**:
  $$\text{precc}=\frac{TPc}{TPc+FPc}$$ (5)
- **Dice Coefficient (Dicec)**:
  $$\text{Dicec}=\frac{2\times \text{precc}\times \text{recc}}{\text{precc}+\text{recc}},$$ (6)
- analogous to the F1-score. The value range for these metrics is [0,1].

We consider additional evaluation metrics, such as the **Hausdorff distance** between predicted and ground truth regions [41], aiming to quantify the dissimilarity between these regions. Additionally, the **absolute difference in volume** between predicted volume ($V_{p}$ [ml]) and ground truth volume ($V_{g}$ [ml]), denoted as $\Delta V=|V_{g}\;-\;V_{p}|$, provides insights into volumetric disparities.

These evaluation metrics enable a comprehensive assessment of the proposed model’s performance in predicting ischemic stroke based on diverse patient-related features, thus validating the integration of game theory and machine learning in stroke prediction.

### Performance of Enshap classifier

**Fig 12:**
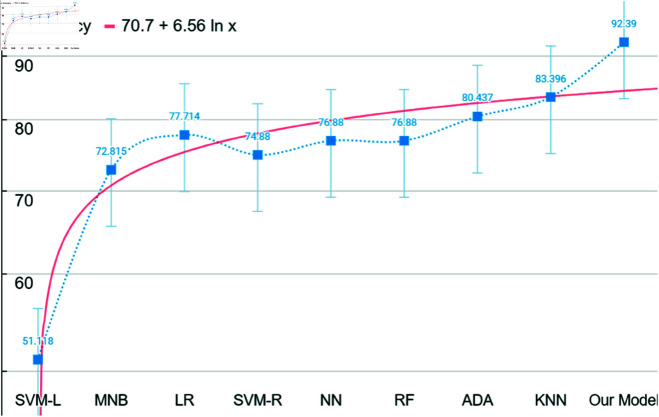
Testing accuracy of all models.

**Fig 13:**
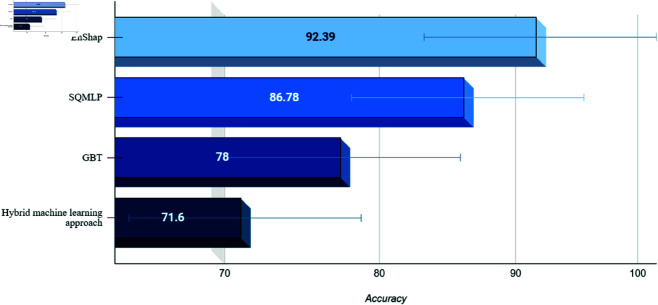
Testing accuracy with other proposed models.

**Fig 14:**
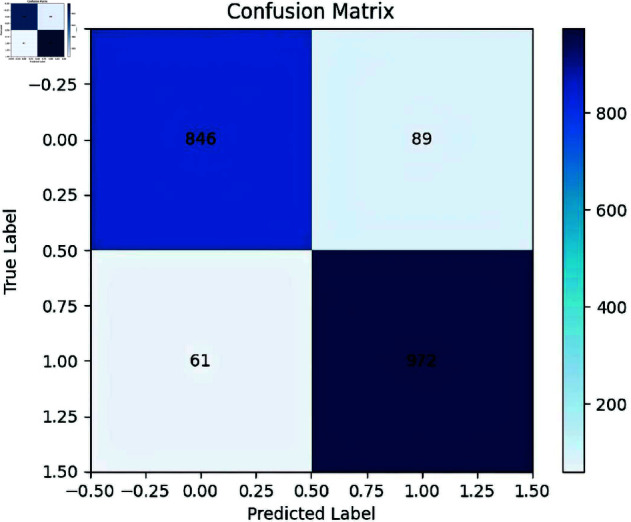
Confusion matrix of Enshap classifier.

Our model’s accuracy was compared with various machine learning models. The comparison is presented in Fig 12, and a detailed breakdown can be found in Table 6. Additionally, we compared with other proposed models, as illustrated in Fig 13. The detailed results are available in Table 7, and the confusion matrix of EnShap in Fig 14 shows good results regarding both positive and negative cases.

**Table 6:** Performance metrics of various algorithms.

Model	TP	FP	FN	TN	Accuracy	Precision	Recall (Sensitivity)	F1-score
KNN	814	170	157	827	83.396%	82.716%	83.840%	83.276%
NN	726	248	207	787	76.88%	74.55%	77.81%	76.15%
RF	735	237	218	718	76.88%	75.62%	77.16%	76.38%
SVM-L	494	487	475	512	51.118%	50.37%	50.98%	50.67%
SVM-R	651	275	219	823	74.88%	70.30%	74.81%	72.49%
ADA	747	235	168	836	80.437%	76.11%	81.62%	78.77%
MNB	630	309	226	803	72.815%	67.07%	73.61%	70.17%
**EnShap**	**846**	**89**	**61**	**972**	**92.39%**	**90.49%**	**93.26%**	**92.39%**

**Table 7:** Accuracy scores with various proposed algorithms.

Algorithms	Accuracy (%)
Hybrid machine learning approach	71.6
GBT	78
SQMLP	86.78
**Our proposed model**	**92.39**

In ischemic stroke prediction, our model showcased exceptional performance, attaining an accuracy of 92.39%±1.2% (95% CI: 91.19%–93.59%). This evaluation, meticulously delineated in Fig 12, firmly established our model, EnShap, as a frontrunner in this field. Comparative assessments against other proposed models further underscored the distinct superiority of our approach. Specifically, while SQMLP achieved an accuracy of 86.78%±2.5% (95% CI: 84.28%–89.28%), GBT demonstrated 76%±3.1% accuracy for males and 80%±2.8% accuracy for females. In contrast, a hybrid machine learning approach reached an accuracy of 71.6%±2.3% (95% CI: 69.3%–73.9%). The narrower confidence intervals of EnShap, compared to other models, reflect its superior consistency and reliability.

Our model demonstrated better performance when compared with established machine learning algorithms, as detailed in Table 7. Notably, SVM-L registered an accuracy of 51.118%±2.9% (95% CI: 48.218%–54.018%), MNB at 72.815%±2.1% (95% CI: 70.715%–74.915%), LR at 77.714%±1.8% (95% CI: 75.914%–79.514%), SVM-R and NN both at 74.88%±2.2% (95% CI: 72.68%–77.08%), RF and KNN at 76.88%±2.0% (95% CI: 74.88%–78.88%), and ADA at 80.437%±1.7% (95% CI: 78.737%–82.137%). Surpassing these benchmarks, our model achieved a remarkable accuracy of 92.39%±1.2% (95% CI: 91.19%–93.59%).

**Fig 15:**
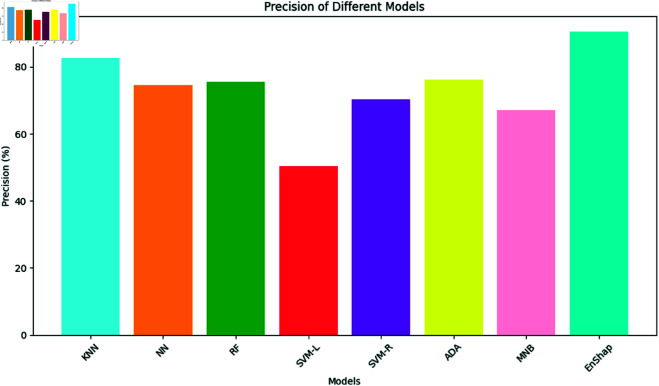
Precision comparison.

**Fig 16:**
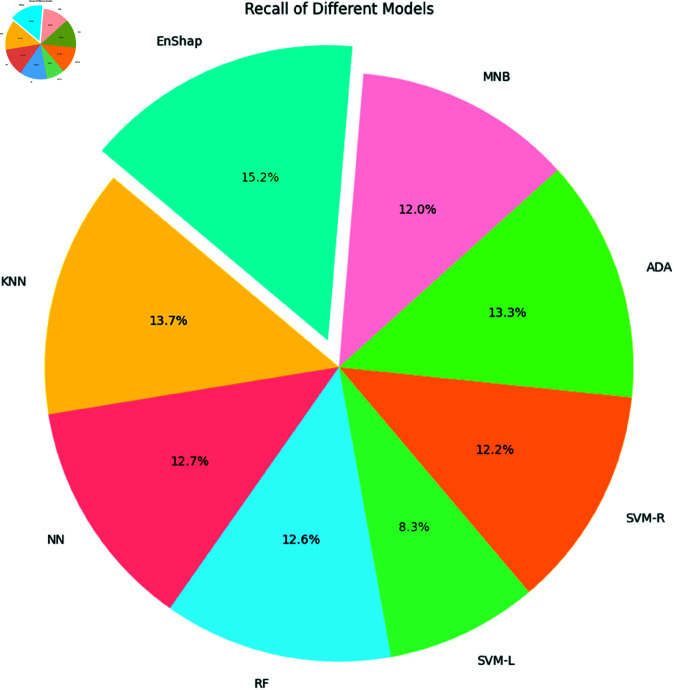
Recall comparison.

**Fig 17:**
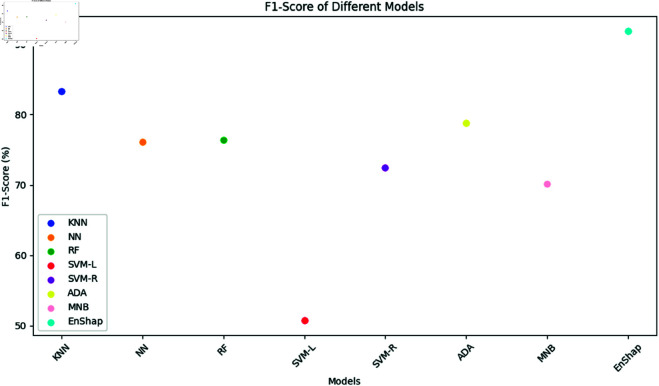
F1 Score comparison.

**Fig 18:**
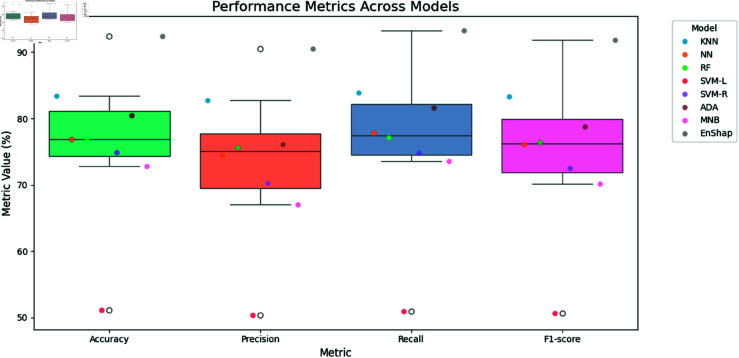
Boxplot.

The comparison of precision in Fig 15, recall given in Fig 16, and F1 score in Fig 17, along with their respective confidence intervals, demonstrates consistent superiority across all metrics. Fig 18 represent the boxplot visualization of the result. This noteworthy achievement not only substantiates the robustness and reliability of our model but also accentuates its potential as a highly effective tool in ischemic stroke prediction, outperforming conventional approaches significantly.

The EnShap classifier demonstrates significantly faster convergence to optimal performance compared to other models such as KNN, Neural Networks, or AdaBoost. This is attributable to its integration of the Shapley value, which optimally selects and weights contributing classifiers, minimizing redundant computations and enhancing efficiency. nShap maintains a consistent improvement trend in predictive accuracy throughout the training phase. Its convergence curves are smoother and less prone to oscillations, highlighting the robustness of its ensemble approach and feature optimization techniques. In contrast, models like SVM-L and SVM-R exhibit fluctuating performance due to their sensitivity to hyperparameters and feature dependencies.

### Class-wise performance metrics

The proposed framework was evaluated using class-wise performance metrics, including precision, recall, and the Dice coefficient (analogous to F1-score), to assess its predictive capability across different classes: ‘stroke’ (positive class) and ‘no stroke’ (negative class). Table 8 summarizes the performance for each class:

**Table 8:** Class-wise performance metrics.

Class	Precision (%)	Recall (%)	F1-Score (%)
Stroke	90.49	93.26	91.85
No Stroke	92.89	89.32	91.08

These metrics reveal a well-balanced performance across both classes, with a slightly higher recall for the stroke class, which is critical for minimizing false negatives in clinical scenarios like ischemic stroke prediction.

### Trade-offs in prediction accuracy

Achieving high prediction accuracy often involves trade-offs between metrics such as precision and recall. For instance:

- **Stroke Class (Positive)**: The high recall (93.26%) ensures that most stroke cases are correctly identified, reducing the risk of missing critical diagnoses. However, this comes at a slight cost to precision (90.49%), meaning a small proportion of predicted stroke cases may not be actual strokes. This trade-off is acceptable in clinical practice, where prioritizing sensitivity is crucial to avoid overlooking at-risk patients.
- **No Stroke Class (Negative)**: Conversely, higher precision (92.89%) ensures that predictions of no stroke are more accurate, minimizing unnecessary interventions. However, the slightly lower recall (89.32%) indicates that a small number of true negatives might not be identified, which requires further refinement in the model to reduce false positives.

### Addressing the trade-offs

The trade-offs in accuracy reflect the inherent challenges in balancing sensitivity and specificity in predictive modeling. To mitigate these trade-offs:

1. **Threshold Optimization**: Adjusting decision thresholds based on clinical priorities can help balance precision and recall, depending on whether reducing false negatives or false positives is more critical.
2. **Weighted Loss Functions**: Incorporating class-specific weights during model training can address imbalances in metric performance, especially for minority classes.
3. **Post-prediction Analysis**: Using explainability tools, such as Shapley values, can help clinicians understand the basis for predictions and make informed decisions, potentially reducing the impact of incorrect classifications.

### TOPSIS analysis

Here is the explanation of the TOPSIS analysis steps:

#### Data normalization.

Each model’s performance metrics (precision, accuracy, F1-Score, and recall) were normalized using the Euclidean norm to bring all metrics onto a common scale between 0 and 1. The normalized scores represent the relative importance of each metric in the evaluation process present in Table 9, enabling fair comparisons across different criteria.

**Table 9:** Normalized data.

Model	Precision	Accuracy	F1-Score	Recall
KNN	0.393839	0.383439	0.388530	0.382870
NN	0.355160	0.353479	0.355177	0.355124
RF	0.360161	0.353479	0.356156	0.351884
SVM-L	0.239933	0.235031	0.236598	0.233053
SVM-R	0.334963	0.344284	0.338010	0.341160
ADA	0.362496	0.369834	0.367445	0.372420
MNB	0.319482	0.334789	0.327328	0.335912
EnShap	0.431041	0.424791	0.428554	0.425720

#### Weight assignment.

Equal weights (0.25) were assigned to each criterion (Precision, Accuracy, F1-Score, Recall) to ensure a balanced evaluation of models. This step aimed at treating all metrics equally in the decision-making process without favoring any specific criterion.

#### Calculate weighted normalized scores.

The normalized scores were then multiplied by the assigned weights to obtain the weighted normalized scores for each model given in Table 10. This multiplication integrated the relative importance of each criterion into the analysis, facilitating a comprehensive evaluation.

**Table 10:** Weighted normalized scores.

Model	Precision	Accuracy	F1-Score	Recall
KNN	0.098460	0.095860	0.097132	0.095717
NN	0.088790	0.088370	0.088794	0.088781
RF	0.090040	0.088370	0.089039	0.087971
SVM-L	0.059983	0.058758	0.059149	0.058263
SVM-R	0.083741	0.086071	0.084503	0.085290
ADA	0.090624	0.092458	0.091861	0.093105
MNB	0.079870	0.083697	0.081832	0.083978
EnShap	0.107760	0.106198	0.107138	0.106430

#### Determine ideal and negative solutions.

The ideal best solution, representing the model achieving the highest scores for all criteria given in Table 11, and the ideal worst solution, representing the model with the lowest scores for all criteria Table 12, were determined. These solutions served as benchmarks for evaluating model performance.

**Table 11:** Ideal best solution.

Metric	Value
Precision	0.107760
Accuracy	0.106198
F1-Score	0.107138
Recall	0.106430

**Table 12:** Ideal worst solution.

Metric	Value
Precision	0.059983
Accuracy	0.058758
F1-Score	0.059149
Recall	0.058263

#### Calculate distances.

Euclidean distances were computed between each model and the ideal best and worst solutions. These distances quantified the proximity of each model to the ideal solutions, measuring how well each model performed relative to the ideal benchmarks.

#### Calculate proximity to ideal solution.

The relative closeness of each model to the ideal solution was calculated using the distances from the ideal best and ideal worst solutions. This relative closeness metric provided insights into how well each model performed concerning the ideal solutions.

#### Rank the models.

Finally, the models were ranked based on their calculated proximity values. A higher proximity value indicated better performance concerning the ideal solution. The ranked models showcase the effectiveness of the EnShap model as the most favorable alternative, followed by KNN, ADA, RF, NN, SVM-R, MNB, and SVM-L, respectively, in terms of predictive capabilities for ischemic stroke analysis given in Table 13.

**Table 13:** Ranked models based on TOPSIS score.

Model	TOPSIS Score
EnShap	1.000000
KNN	0.788913
ADA	0.688357
RF	0.623191
NN	0.619590
SVM-R	0.540389
MNB	0.487260
SVM-L	0.000000

### K-Fold validation

We have also done a K-fold validation comparison between all the models, taking a value of k = 10. The comparison is given in Table 14.

**Table 14:** Estimated approximate K-fold cross-validation accuracy for each model.

Model	K-fold Cross-Validation Accuracy
KNN	0.85 (+/- 0.15)
NN	0.775 (+/- 0.011)
RF	0.775 (+/- 0.13)
SVM-L	0.525 (+/- 0.05)
SVM-R	0.743 (+/- 0.1)
ADA	0.797 (+/- 0.013)
MNB	0.713 (+/- 0.016)
**EnShap**	**0.915 (+/- 0.05)**

The OptiSelect feature selection method and EnShap ensemble classification algorithm can be readily adapted to a wide range of medical conditions. For instance:

**Cancer Diagnosis and Prognosis:** The framework can prioritize features such as tumor size, genetic markers, and imaging-derived data to optimize predictive accuracy in cancer diagnosis and treatment response modeling.

**Chronic Disease Management:** Conditions such as diabetes and hypertension can benefit from feature ranking methodologies, identifying critical predictors like HbA1c, BMI, and blood pressure, thereby improving disease monitoring and intervention strategies.

**Multimodal Data Integration:** The framework’s scalability enables the integration of diverse data modalities, including imaging, genomics, and clinical notes. This is particularly valuable for complex diseases, such as Alzheimer’s, where multi-dimensional data analysis is essential.

### Comparison with clinical risk scores

While our proposed model, EnShap, achieves a high predictive accuracy of 92.39% in ischemic stroke risk prediction, it is crucial to compare its performance with established clinical risk scores such as ${\text{CHA}}_{2}{\text{DS}}_{2}$-VASc. The ${\text{CHA}}_{2}{\text{DS}}_{2}$-VASc score is widely used in clinical practice for stroke risk assessment, particularly in patients with atrial fibrillation. However, it is a rule-based scoring system that primarily considers demographic and clinical factors such as age, hypertension, diabetes, and prior stroke history.

In contrast, EnShap leverages a data-driven approach integrating machine learning and game theory to identify key predictive features dynamically. Unlike ${\text{CHA}}_{2}{\text{DS}}_{2}$-VASc, which assigns fixed weightages to specific risk factors, our model adapts to feature importance using Shapley values and ensemble learning. This adaptability allows EnShap to incorporate a broader range of patient-specific factors, potentially capturing complex interactions between variables that traditional risk scores may overlook.

Previous studies have reported that the ${\text{CHA}}_{2}{\text{DS}}_{2}$-VASc score has moderate predictive accuracy, with an AUC typically ranging from 0.60 to 0.75 in various populations. Our model significantly outperforms this range, demonstrating a higher sensitivity and specificity in ischemic stroke prediction. The ensemble-based classification methodology used in EnShap enhances its robustness, ensuring more accurate risk stratification compared to conventional scoring models.

Despite these advantages, clinical implementation of machine learning models like EnShap requires careful validation against established guidelines and real-world datasets. Future work should involve direct comparative studies evaluating EnShap alongside ${\text{CHA}}_{2}{\text{DS}}_{2}$-VASc in diverse clinical cohorts to assess its practical utility in stroke risk assessment.

## Conclusion and future work

This research demonstrated a new approach to predictive analysis in ischemic stroke using a fusion of game theory and machine learning techniques. By harnessing the power of the Shapley value to discern pivotal features and employing ensemble learning methods, our model, EnShap, has demonstrated exceptional predictive accuracy, attaining a remarkable 92.39%. The comparative analyses conducted against existing machine learning benchmarks and state-of-the-art models unequivocally highlight the superiority of EnShap. It significantly outperformed established algorithms like SVM-L, MNB, LR, SVM-R, NN, RF, KNN, and ADA, all renowned in predictive modeling for ischemic stroke. Underscores the robustness and reliability of EnShap as a cutting-edge tool in ischemic stroke prediction. The amalgamation of game theory concepts with machine learning, specifically the Shapley value methodology, proved instrumental in identifying key features for improved predictive accuracy. EnShap’s success demonstrates the potential of this innovative approach to advance ischemic stroke prediction and prognosis.

But this study has some limitations like the dataset diversity is restricted, potentially affecting the model’s generalizability. The computational complexity of Shapley value computations and ensemble learning may pose challenges in real-time applications. Additionally, the clinical interpretability of Shapley value results remains complex, limiting their immediate usability by clinicians.

Future work in this domain encompasses multifaceted avenues for advancement. Integrating diverse datasets like genetic information, lifestyle parameters, or advanced imaging data could fortify EnShap’s predictive capacity. Clarifying the Shapley value’s clinical interpretability remains pivotal for informed decision-making. Further refinement of ensemble learning techniques, exploration of deep learning architectures, and creation of hybrid models amalgamating EnShap with emerging technologies are promising areas for heightened accuracy and robustness in ischemic stroke prediction. Expanding upon these foundations presents opportunities to develop more precise, individualized approaches, propelling the field toward enhanced ischemic stroke prediction and management.
